# Magnetic resonance images and measurements of the volume, proportion, and longitudinal distribution of contractile and non-contractile tissue in the dorsi- and plantar flexor muscles of healthy young and older adults

**DOI:** 10.1186/s13104-018-4026-x

**Published:** 2018-12-20

**Authors:** Christopher J. Hasson, Jane A. Kent, Graham E. Caldwell

**Affiliations:** 1 Departments of Physical Therapy, Movement and Rehabilitation Sciences, Bioengineering, and Biology, Northeastern University, Boston, MA USA; 2 Department of Kinesiology, University of Massachusetts Amherst, Amherst, MA USA

**Keywords:** MRI, Cross-sectional area, Contractile tissue, Non-contractile tissue, Dorsiflexor, Plantar flexor, Volume, Aging, MATLAB

## Abstract

**Objective:**

This paper presents magnetic resonance images of the dorsi- and plantar flexor muscles for individual young and older healthy adults. Also included are measurements of the volume, proportion, and longitudinal distribution of contractile and non-contractile tissue. This dataset was previously used to quantify age-related differences in these measures, constrain subject- and muscle-specific estimates of dorsi- and plantar flexor maximal isometric force capability, and quantify the degree to which maximal isometric force capability explains the age-related variance in postural control.

**Data description:**

The data include contiguous axial magnetic resonance images of the lower leg for 12 young (21–31 years) and 12 older (66–79 years) healthy adults. The data are in the form of MATLAB binary files with a freely distributable custom MATLAB analysis program that allows image viewing and navigation in two and three dimensions, muscle outlining, tissue segmentation, and cross-sectional area calculation. The latter measurements are contained in a set of companion MATLAB binary files, which are included with the image data files. If desired, the magnetic resonance images could be used to identify other anatomical structures, or the MATLAB programs could be used to analyze other image sets.

## Objective

Magnetic resonance (MR) images were collected to measure the volume, proportion, and longitudinal distribution of contractile and non-contractile tissues in the dorsi- and plantar flexor muscles in healthy young and older adults. A custom MATLAB (R2018a; Mathworks, Natick MA) program, which is included with the dataset [1], was created to view the images, analyze the data, examine potential age-related differences in these measures, and quantify reliability. As reported in Hasson et al. [2], older adults had reduced muscle volumes with a higher proportion of non-contractile tissue, indicating a loss of muscle quantity and quality. The dataset also describes the distribution of intramuscular non-contractile tissue, which may have clinical relevance (e.g., intramuscular fat has been linked to insulin sensitivity [3]). Contractile volume is highly correlated to the maximal isometric force capability (*P*_0_) of an individual muscle [4], which cannot be determined from joint torque measurements alone when there is more than one active muscle crossing a joint [5]. Hasson and Caldwell [6] addressed this redundancy challenge by combining the MRI-derived contractile volumes with ultrasound measurements, joint torque assessments, and numerical optimization to derive subject- and muscle-specific estimates of dorsi- and plantar flexor *P*_0_, length and velocity dependent muscular properties, and series-elastic stiffness. Older adults had reduced *P*_0_, altered force–length properties, slower force–velocity characteristics, and stiffer series-elasticity. Using the same participants, a related study by Hasson et al. [7] found that age-related changes in these muscle properties explained about 50–60% of the variance in measures of postural control. Since muscle properties can be altered with resistance training, even in the elderly [8], it is conceivable that muscular training could improve postural control in older adults. The dataset presented here could have continued utility for understanding age-related changes in muscle physiology and associated impacts on motor function and overall health.

## Data description

### Subjects

The data are from 12 young (21–31 years) and 12 older (66–79 years) adults. Each age group had an equal number of males and females. All were free from musculoskeletal or neurological impairments, engaged in regular physical activity, and were independent community-dwelling adults. More information about the subjects, such as height, weight, etc. can be found in Table 1 of Hasson et al. [2].

**Table 1:** Overview of data files/data set

Label	Name of data file/data set	File types	Data repository
MRI analysis program	Main analysis program code (MRI_Process) and associated functions and files to process MRI data binaries Associated files: MRI_Process.m MRI_Process.fig Help_Image_Data.mat GNU General Public License.txt draggable.m CJ_detrend_and_filter.m User Guide.pdf	MATLAB scripts (.m), MATLAB GUI (.fig), ASCII files (.txt), MATLAB binaries (.mat), and PDFs	figshare 10.6084/m9.figshare.c.4270805
Sequential MRI data and analysis output files	MRI data binaries for all subjects, labeled: XXN_MRI_Data_Combined.mat Where XX is the group identifier (YF = young female; YM = young male; OF = old female; OM = old male) and N is the subject number (1–6) Folder also contains results of muscle boundary identification, tissue segmentation, and cross-sectional area calculations; file name has “Output” appended, e.g. XXN_MRI_Data_Combined_Output.mat Other files: Output Variables Description.pdf (Description of data in the output files.) File_Naming_Convention.pdf (self-explanatory)	MATLAB binaries (.mat) and PDFs	

### Data collection and processing

A 1.5 T magnetic resonance imaging (MRI) system (Sigma EchoSpeed Plus, General Electric) was used to capture a contiguous series of axial MR images from the left leg of each subject (phased-array coil, T1-weighted spin echo sequence, 4 mm “slice” thickness (no gaps), 400 ms repetition time, 11 ms echo time, 512 × 512 pixel resolution, and 30 cm field of view. The raw MR files were in DICOM (Digital Imaging and Communications in Medicine) format. MATLAB was used to convert each subject’s set of DICOM image files (reflecting multiple slices) into a single MATLAB binary data file (with.*mat* extension). These MATLAB binary files are available in the data repository (Sequential MRI Data; Table 1). Each file contains a 512 × 512 × $ns$ matrix of 16-bit integers representing grayscale pixel intensities, where $ns$ is the number of slices (axial images) for a subject. Each binary file also contains a data structure with information about the image height and width and pixel spacing $s$ (the physical distance between the centers of adjacent pixels). The MATLAB binaries duplicate the DICOM image data but eliminate identifying information stored in the DICOM files; therefore, only the MATLAB binaries reside in the data repository to protect participant privacy.

### Analysis program

A custom MATLAB program was created to read in the MATLAB binary files, view the MRI images, identify muscle cross-sectional areas (CSAs), separate contractile and non-contractile tissue, and calculate the muscle volumes and visualize them in 3D. This program was updated to be compatible with MATLAB R2018a, and the source code and user guide are available in the data repository (see MRI Analysis Program and User Guide; Table 1). If one wishes to convert other DICOM image sets into MATLAB binaries the MATLAB function “dicomread.m” (MATLAB Image Processing Toolbox) can be used.

### Muscle cross-sectional area

As reported in Hasson et al. [2] the perimeters of the soleus, lateral and medial heads of the gastrocnemius, and the combined dorsiflexor muscles (tibialis anterior, extensor hallucis longus, extensor digitorum longus, and peroneus tertius) were outlined in every other slice using the MATLAB analysis program. The program calculates the CSA of each muscle in each identified slice and separates the CSA into contractile vs. noncontractile based on pixel intensity. For each subject, the identified leg and muscle boundaries, pixel intensity data, and CSAs are saved in an output file, which is automatically read and displayed when the MATLAB analysis program is executed. These files are in the data repository (analysis output files; Table 1).

## Limitations

The data represent a limited sample of relatively healthy young and older adults and may not be representative of other populations. Although manually outlining muscles in MR images is subject to human error, the measured data showed good reliability with inter-class correlation coefficients above .90 for intra- and inter-observer reliability (see details in [2]).

## Abbreviations

CSA: cross-sectional area
DICOM: digital imaging and communications in medicine
MRI: magnetic resonance image
*P*_0_: maximal isometric muscle force capability
